# Editorial: Methods and applications in emotion science

**DOI:** 10.3389/fpsyg.2022.1058322

**Published:** 2022-11-14

**Authors:** Lucy J. Troup, Wenhai Zhang

**Affiliations:** ^1^Division of Psychology, University of the West of Scotland, Paisley, United Kingdom; ^2^College of Educational Science, Hengyang Normal University, Hengyang, China; ^3^Shanghai Key Laboratory of Mental Health and Psychological Crisis Intervention, School of Psychology and Cognitive Science, East China Normal University, Shanghai, China

**Keywords:** emotion faces, fMRI, implicit processing, social anxiety, methods in emotion science, cognitive reappraisal, artificial intelligence (AI), applications in emotion science

Emotion science is associated with a broad spectrum of methods and applications (deGelder, 2017). This Research Topic brings together innovative approaches and applications of our current understanding in this field. The present collection reflects the interdisciplinary reach of this area of research in psychology.

At the forefront of methods and applications is the use of cognitive reappraisal and its effect on emotion processing (Gross and Thompson, 2014). Shigematsu and Kobayashi, in “*Relationship Between Emotion Regulation Strategies and Total Conviction in Promoting Behavior Change*,” address the context in which cognitive reappraisal is presented and how it might have an effect on emotion regulation. They utilized a cold pressor task which was presented in the context of “total conviction,” encouraging cognitive reappraisal of the task to a positive outcome, i.e., the task would lead to an improvement in health. Despite the fact that they did not find the conviction to be a significant predictor of reappraisal, it was nonetheless a novel approach to addressing the role of re-evaluation in cognitive appraisal.

Similarly, cognitive reappraisal plays an important role in reducing possible harm from negative emotions (Dryman and Heimberg, 2018). As one of the basic human emotions, sadness is characterized by an evolutionary response to loss, or when a goal is not achieved. Persistent sadness has the potential to develop into a psychiatric disorder, which makes the regulation of sadness instrumental in avoiding such a negative outcome (e.g., Zilverstand et al., 2017). Yan et al. found that down-regulation reappraisal significantly reduced subjective feelings of sadness, but expressive suppression did not. Moreover, they used event-related potentials to reveal that reappraisal (300–1,500 ms after image onset) and expressive suppression (300–600 ms) significantly weakened the late positive potential (LPP) induced by sadness. This suggests that down-regulation reappraisal is a more effective strategy in regulating sadness relative to expressive suppression.

The assessment of emotion processing in the context of “culture-free” brief psychiatric tools is addressed by Ali et al. Based on the tripartite model of psychopathology, the Depression, Anxiety, and Stress Scale-8 (DASS-8) described by their contribution showed excellent psychometric properties, invariant at the configural and metric levels across all countries included in its development. They demonstrated the DASS-8 can be successfully used as an initial screen for depression and anxiety disorders in English-speaking and African cultures.

Social networking has changed how people conduct relationships. It has also contributed to the development of new methods for investigating behavior (Cheng et al., 2019). Ricciardi et al. in their manuscript used social network analysis theory to investigate how dynamic social relationships are influenced by emotion regulation, importantly bringing together a new perspective, network science theory, and social network analysis to understand emotion regulation in complex social settings.

As a reflection of the diversity of work on this special topic, Kutsuzawa investigated the significance of the use of emojis representing the emotional expression of faces across a range of age groups in their manuscript “*Age Differences in the Interpretation of Facial Emojis: Classification on the Arousal-Valence Space*.” Using a categorization in a valence/arousal space, there were similar clusters around six emotional expressions for all age groups. Interestingly, however, negative emotional expression led to greater arousal in middle-aged adults compared to younger adults.

Meaningful developments in applications of emotion science show that feelings play an important role in business decision-making activities (Shrestha et al., 2019). Lu et al. used VOSviewer software with a bibliometric analysis method to comprehensively organize the emotional research literature in the field of entrepreneurship. It was observed that the current research hotspots can be divided into five categories: emotions and entrepreneurship among college students; family emotions and entrepreneurship; the role of emotions in successful entrepreneurship; emotions in the context of entrepreneurial failure; and entrepreneurial passion. This work provides a foundation for developing applications of emotion science in everyday life.

Emotion recognition enables the affective Brain Computer Interface (aBCI) to accurately perceive the states of brains (Shanechi, 2019). A tool that has had a significant impact on this area of research is the EEG-based aBCI. It has developed into a widely used application for emotion decoding, including machine learning and deep learning. Based on AConvNet, Liang et al. proposed a novel nuclear Norm Regularized Deep Neural Network (NRDNN) framework that can learn the high-level representations of EEG signals across the brain. NRDNN can capture the structural information among different brain regions in EEG decoding and improve the interpretation of affective EEG classification performance.

Another AI application explored how humanoid robots and androids exhibit human-like emotional appearances (see Stock-Homburg, 2022 for a review). Sato et al. developed an android head called Nikola as a tool for testing live face-to-face emotional interactions. Nikola produced single facial actions centered on prototypical facial expressions for six basic emotions as well as dynamic facial expressions for those emotions at four different speeds. Tools like Nikola have important applications in psychological experiments, examining face-to-face emotional interactions with high ecological validity and control. They have the potential to transmit emotional messages to humans, which may be useful in a wide range of applied situations, e.g., elder care, behavioral interventions, and customer service.

In sum, this Research Topic of manuscripts represents the highly interdisciplinary nature of the current state of methods and applications in emotion science.

## Author contributions

LT and WZ contributed equally to the writing of this editorial. Both authors contributed to the article and approved the submitted version.

## Funding

This topic was funded by the Hunan Natural Science Foundation of China (2022JJ30099), the Research Project of Shanghai and Technology Commission (20dz2260300), and the Fundamental Research Funds for the Central Universities.

## Conflict of interest

The authors declare that the research was conducted in the absence of any commercial or financial relationships that could be construed as a potential conflict of interest.

## Publisher's note

All claims expressed in this article are solely those of the authors and do not necessarily represent those of their affiliated organizations, or those of the publisher, the editors and the reviewers. Any product that may be evaluated in this article, or claim that may be made by its manufacturer, is not guaranteed or endorsed by the publisher.
